# Development of Pullulan-Based Colorimetric Film with Butterfly Pea for Shrimp Freshness Monitoring

**DOI:** 10.3390/foods15213959

**Published:** 2026-11-04

**Authors:** Karolina Kraśniewska, Kevin Rakoto, Małgorzata Gniewosz

**Affiliations:** Department of Food Biotechnology and Microbiology, Faculty of Bioeconomy and Food Systems, Warsaw University of Life Sciences—SGGW (WULS—SGGW), Nowoursynowska Str., 159c, 02-776 Warsaw, Poland; kevin.rakoto@o2.pl

**Keywords:** shrimp, freshness indicator, pullulan colorimetric film, butterfly pea flowers

## Abstract

Colorimetric indicators based on biopolymers and natural colorants represent a promising approach for modern food freshness monitoring systems. In this study, colorimetric films based on pullulan with 2% and 4% butterfly pea flower (BPF) powder were fabricated and evaluated for their physical and optical properties. Furthermore, visual and colorimetric analyses of the films color changes in response to pH and ammonia were also performed. Finally, film with 4% of BPF were tested as a freshness-monitoring label during shrimp storage. The results showed that BPF incorporation did not significantly affect film thickness, but it increased film opacity and improved UV and visible-light barrier properties. The incorporation of BPF also altered and intensified the color of the pullulan films. The films exhibited a distinct response to pH changes, with their color changing from red under strongly acidic conditions, through purple and blue, to olive green under alkaline conditions. Moreover, the films responded to ammonia, exhibiting a visually perceptible color change from blue to green. The colorimetric film containing 4% BPF exhibited visually distinct color changes associated with shrimp spoilage, indicating its potential as a freshness-monitoring label during refrigerated storage.

## 1. Introduction

Shrimp are widely consumed seafood products with high nutritional value, a characteristic taste, and a delicate texture. However, due to their high moisture and protein content, shrimp are highly perishable [1,2]. Therefore, refrigerated or frozen storage is commonly used to maintain shrimp quality and extend their shelf life. Nevertheless, even under these storage conditions, gradual changes in quality occur, leading to a loss of freshness and product spoilage [3]. Shrimp quality degradation is mainly associated with endogenous enzymatic activity, the activity and proliferation of spoilage microflora, and oxidative reactions [3,4]. In particular, microbial spoilage of shrimp leads to the formation of volatile basic compounds such as trimethylamine, dimethylamine, and ammonia, included as a components of total volatile basic nitrogen (TVB-N) [5]. The accumulation of these compounds is associated with an increase in pH, as well as unpleasant sensory changes, particularly in the taste and odor of shrimp [5,6].

Therefore, colorimetric pH indicators in the form of intelligent films that can be integrated into the packaging have become important tools for real-time monitoring of food quality and spoilage during storage and distribution [7]. They can provide information on shrimp freshness without opening the package and, importantly, allow consumers to independently assess product quality [2].

Colorimetric pH indicators generally consist of two main components: a polymeric matrix and a pH-sensitive compound incorporated into the matrix [8,9]. So far, various polysaccharide- or protein-based biopolymers have been used as the base matrix, while natural colorants such as anthocyanins, betalains, and curcumin have been studied as pH-sensitive compounds [10,11,12].

Among polysaccharide-based materials, pullulan provides a suitable film-forming matrix for use as a colorimetric pH indicator [13]. Pullulan is an extracellular polysaccharide produced by *Aureobasidium pullulans* [14]. This compound is water-soluble, tasteless, odorless, and biodegradable. Moreover, pullulan possesses the ability to form thin films, which are transparent and colorless and exhibit good oxygen barrier properties [15,16]. Pullulan also exhibits food-grade properties, has GRAS status, and is approved as a food additive (E1204) in the European Union [17,18,19]. This biopolymer can serve as a suitable matrix for the incorporation of functional compounds. So far, various bioactive substances, including natural antimicrobial compounds such as essential oils or plant extracts, have been incorporated into pullulan-based films to enhance their functional properties and develop active packaging systems for extending the shelf life of food [17,20,21,22]. Beyond its use in active packaging, pullulan also has potential as a polymer matrix for developing intelligent colorimetric films [23].

Among natural colorants, anthocyanins have received considerable attention as pH-sensitive compounds for developing colorimetric indicators. Anthocyanins are water-soluble polyphenolic pigments widely distributed in plants, where they are responsible for the red, pink, purple, and blue colors of many flowers, fruits, and vegetables [24]. An important characteristic of anthocyanins is their ability to exhibit distinct and visible color changes in response to changes in pH [25]. Therefore, anthocyanins can serve as natural pH-sensitive components in intelligent films for monitoring food freshness, since microbial activity during food spoilage can lead to the formation of volatile amines and/or organic acids, which are accompanied by changes in pH [26,27]. An interesting source of anthocyanins is the flower of *Clitoria ternatea* L., also known as butterfly pea flower (BPF), which are characterized by an intense blue color. This characteristic coloration of the flowers is mainly due to the presence of ternatins, anthocyanins that belong to the group of polyacylated delphinidin derivatives [28,29]. BPF is traditionally used as a natural blue colorant for rice in the Malaysian dish nasi kerabu and as a coloring agent in beverages. The flowers are also used to prepare herbal tea in Southeast Asia [30,31]. BPF anthocyanins exhibit an intense and saturated color [30]. At the same time, these compounds exhibit high pH sensitivity and show distinct color changes across a wide pH range [32]. So far, several studies have reported the use of anthocyanins from butterfly pea flowers to produce intelligent colorimetric films for monitoring food freshness. For example, Ahmad et al. [33] fabricated a sago starch-based film with butterfly pea anthocyanins as a pH-sensitive indicator for monitoring the freshness of chicken meat. Meanwhile, Narayanan et al. [34] developed a gelatin/pectin-based colorimetric indicator using butterfly pea flower extract to monitor fish freshness during refrigerated storage. Also, sodium alginate films incorporated with *C. ternatea* anthocyanin-rich extract showed promise as a tool for monitoring the freshness of milk, pork, and shrimp [35].

The concept of this study was to use butterfly pea flowers as a colorimetric indicator in a pullulan-based biopolymer matrix to monitor changes in freshness in fresh shrimp. Although the use of such an indicator is not novel, to the best of the authors’ knowledge, most studies on the application of butterfly pea flowers as natural pH-sensitive indicators have focused on anthocyanin-rich extracts [33,34,35]. In contrast, this study presents the possibility of using commercially available butterfly pea flower powder directly, without an additional extraction step. Although this approach may simplify film preparation by eliminating the need to select extraction solvents and conditions, it may also present certain limitations in terms of pigment dispersion, optical uniformity, and material costs.

The aim of this study was to characterize a pullulan film incorporating BPF powder and evaluate its potential as a colorimetric freshness-monitoring label for shrimp packaging.

## 2. Materials and Methods

### 2.1. Materials and Reagents

Pullulan was purchased from Hayashibara Co. (Okayama, Japan). Commercially available pre-ground butterfly pea flower (BPF) powder (*Clitoria ternatea* L.) was purchased from Ambasada Herbaty (Warsaw, Poland) and used without further processing. Buffer solutions (pH 1–12), glycerol, boric acid and sodium hydroxide (NaOH) were purchase from Chempur (Piekary Śląskie, Poland). A 25% ammonia solution was obtained from POCH (Gliwice, Poland), and percholic acid solution, hydrochloric acid (HCl), and indicators: phenolphthalein and Tashiro were purchased from Warchem (Zakręt, Poland). Microbiological medium Plate Count Agar (PCA) was purchased from BTL (Łódź, Poland).

### 2.2. UV-VIS Spectroscopy Measurements

To assess the pH sensitivity of BPF powder, 50 mg of BPF was dissolved in 25 mL of each buffer solution at pH values ranging from 1 to 12. Changes in the color of the BPF solutions were visually observed and photographed using a smartphone camera in a light tent (Puluz, LED 5500 K, Shenzhen PULUZ Technology Limited, Shenzhen, China).

Additionally, the UV-Vis absorbance spectra of each solution were measured from 400 to 700 nm using a UV–Vis spectrophotometer (BioSpectrometer, Eppendorf, Hamburg, Germany).

### 2.3. Preparation of Pullulan Films

A base pullulan film-forming solution containing 15% (*w*/*v*) pullulan and 4% (*w*/*v*) glycerol was prepared in distilled water and heated at 80 ± 2 °C. Subsequently, BPF powder was incorporated into the base solution, previously cooled to room temperature, to obtain final concentrations of 2% (*w*/*v*) and 4% (*w*/*v*), and the mixture was then stirred for 15 min at 500 rpm using a magnetic stirrer (IKA, Warsaw, Poland). After that, film-forming solutions (10 mL) were cast onto Petri dishes (Ø 90 mm) and dried in an oven (OVEN, BEING Scientific, Ontario, CA, USA) at 35 °C for 24 h. The control pullulan film and the films containing 2% and 4% (*w*/*v*) BPF powder were designated as P_C, P + BPF_2, and P + BPF_4, respectively.

### 2.4. Characterization of Film

#### 2.4.1. Thickness

The thickness of the films (P_C, P + BPF_2, and P + BPF_4) was measured using a thickness gauge (BYKO-Test 4500, BYK-Gardner, Geretsried, Germany). Measurements were performed at randomly selected locations across each film sample.

#### 2.4.2. Optical Properties (Opacity and Light Transmittance)

The opacity and light transmittance of the films (P_C, P + BPF_2, and P + BPF_4) were determined using a UV–Vis spectrophotometer (UV–VIS SP-8001, Metertech Inc., Taipei, Taiwan). Film samples (1 × 4 cm) were placed directly into the spectrophotometer sample holder, and the absorbance was measured at 600 nm (Abs_600). Film opacity was calculated according to Equation (1):

(1) $$\mathrm{Opacity}\;=\;\frac{{Abs}_{600}}{t}$$

where *Abs*_600_ is the absorbance value at 600 nm; *t* is the film thickness (mm).

The UV barrier and visible light transmission properties were evaluated by measuring the transmittance at 280 and 600 nm, respectively. The results were expressed as the percentage (%) of light transmittance.

#### 2.4.3. Evaluation of Film Sensitivity to pH

The color response of films to different pH values was evaluated using buffer solutions with pH values ranging from 1.0 to 12.0. Film samples were cut into 3.5 × 3.5 cm pieces and immersed in the pH solutions for 1 min. After immersion, the color parameters of films were measured using a colorimeter (Chroma Meter CM-5, Konica Minolta, Osaka, Japan). The color parameters (L*, a*, and b*) were recorded. The total color difference (Δ*E*) was calculated according to Equation (2) using the color parameters of the film before immersion as the reference.

(2) $$\Delta E=\sqrt{{\left({L*}_{c}-{L*}_{e}\right)}^{2}+{\left({a*}_{c}-{a*}_{e}\right)}^{2}+{\left({b*}_{c}-{b*}_{e}\right)}^{2}}$$

where L*_c_, a*_c_, b*_c_ are values for the control film samples, L*_e_, a*_e_, b*_e_ are values for the experimental film samples.

Additionally, changes in the color of films exposed to different pH levels were visually observed and photographed with a smartphone in a light tent (Puluz, LED 5500 K, Shenzhen PULUZ Technology Limited, Shenzhen, China).

#### 2.4.4. Evaluation of Film Sensitivity to Ammonia (NH_3_)

The color response of films to ammonia (NH_3_) was evaluated using the method described by Boonsiriwit et al. [36]. With modification. solutions of ammonia at three concentrations: 0.1 M, 0.5 M, and 1 M. The containers containing ammonia solutions (20 mL) were covered with lids with the film pieces (3.5 × 3.5 cm) attached to their inner side. The distance between the surface of the ammonia solution and the film was maintained at 2 cm. The color parameters of films after 0, 10, 20, 40, 50, 60, 80, 100, 120, 140, 160, and 180 min of exposure to ammonia vapor were measured using a colorimeter (Chroma Meter CM–5, Konica Minolta, Osaka, Japan). The color parameters (L*, a*, and b*) and hue angle (*h*°) were recorded.

The total color difference (Δ*E*) was calculated according to Equation (2) using the color parameters of the film before exposure to ammonia as the reference. Changes in the color of the films exposed to different ammonia concentrations were also visually observed and photographed using a smartphone camera in a light tent (Puluz, LED 5500 K, Shenzhen PULUZ Technology Limited, Shenzhen, China).

#### 2.4.5. Determination of Film Color Stability 

The color stability of the films was evaluated over 21 days of storage under the following conditions: 4 ± 1 °C and RH 70 ± 2% in the dark; 23 ± 1 °C and RH 50 ± 2% in light; and 23 ± 1 °C and RH 50 ± 2% in the dark. The color parameters of the films were measured at selected time points during storage. The hue angle (*h*°) was recorded, and the total color difference (Δ*E*) was calculated using Equation (2) to assess the color stability of the films.

### 2.5. Monitoring Shrimp Freshness by Colorimetric Films

Whole shrimp (*Litopenaeus vannamei*) were purchased from a local market and stored at 4 °C for no longer than 3 h before use. Shrimp weighing approximately 25 g were selected and placed into transparent polypropylene (PP) containers (110 × 80 × 50 mm) with lids. The containers were then sealed with lids to which intelligent films had previously been attached on the inner side. The film samples placed on the lids measured 3.5 × 3.5 cm. As a control, PP containers sealed with colorimetric films but without shrimp were prepared and stored under the same refrigerated conditions (4 °C) for 5 days.

The prepared samples were stored under refrigerated conditions (4 °C) for 5 days, and analyses were performed on days 1, 2, 3, 4 and 5. At each time point, five (*n* = 5) randomly selected packages with films were used for the following analyses: analysis of film color, determination of total volatile basic nitrogen (TVB–N) content, microbiological analysis, and pH measurement of shrimp.

#### 2.5.1. Color Analysis of Intelligent Film

The color parameters of the film were measured using a colorimeter (Chroma Meter CM-5, Konica Minolta, Osaka, Japan). Before color measurement, the film were not detached from the lids and the color measurements were performed with the film attached to the lids. The color parameters (L*, a*, and b*) and hue angle (*h*°) were recorded. The total color difference (Δ*E*) was calculated using Equation (2), with the color parameters measured on day 1 as the reference.

The color stability of the films in the control packages without shrimp was also evaluated before and after the 5-day storage period.

#### 2.5.2. Total Volatile Basic Nitrogen Analysis

Total volatile basic nitrogen (TVB–N) was determined according to the protocol described in Commission Regulation (EC) No 2074/2005 [37]. A shrimp sample (10 g) was homogenized with 90 mL of 6% perchloric acid and subsequently filtered. Then, 50 mL of the filtrate, neutralized with 20% NaOH, was distilled using a KjelFlex K-360 apparatus (BÜCHI, Flawil, Switzerland) into a receiver containing 3% boric acid. The distillate was titrated with 0.01 M HCl using Tashiro indicator until a pink color was obtained. The content of TVB–N was calculated according to Formula (3):

(3) $$TVB-N\;(mg\;N/100\;g\;samples)=\frac{\left(Vs-Vb\right)\times 0.14\times 2\times 100}{m}$$

where Vs—volume of 0.01 M HCl solution used for titration sample (mL), Vb—volume of 0.01 M HCl solution used for titration blank sample (mL), m—weight of sample [g].

#### 2.5.3. pH Measurement

The pH of the shrimp was measured using a portable pH meter with a penetration probe (Testo 206-pH1, Testo SE & Co. KGaA, Titisee - Neustadt, Germany), which was inserted into the shrimp muscle tissue at different locations to calculate the mean value.

#### 2.5.4. Microbiological Analysis

Shrimp samples (5 g) were transferred into a stomacher bag and mixed with 45 mL of physiological saline. The samples were homogenized using a Stomacher (Stomacher 400 Circulator, Seward Ltd., Worthing, UK,). Subsequently, a series of 10-fold decimal dilutions was prepared in physiological saline. The total number of aerobic microorganisms was determined using the pour-plate technique in accordance with EN ISO 4833-1:2013 [38]. Briefly, 1 mL of the appropriate dilution of each shrimp sample was transferred into a sterile Petri dish and mixed with PCA. The samples were then incubated at 30 °C for 48–72 h. Results were expressed as colony-forming units per gram of sample (CFU/g) and converted to log CFU/g.

### 2.6. Statistical Analysis

All experiments were performed at least three times, and the results were expressed as mean ± standard deviation (SD). Statistical analysis was performed using Statistica software (version 13.3.PL, TIBCO, Palo Alto, CA, USA). The data were analyzed using one-way analysis of variance (ANOVA), followed by Tukey’s post hoc test. Differences were considered statistically significant at *p* < 0.05. Spearman’s rank correlation analysis was performed to assess the relationships between shrimp quality parameters (TVB–N, pH, and TMC) and film color parameters (Δ*E* and hue angle (*h*°)).

## 3. Results and Discussion

### 3.1. Color Response and Absorption Spectra of BPF

**Figure 1:**
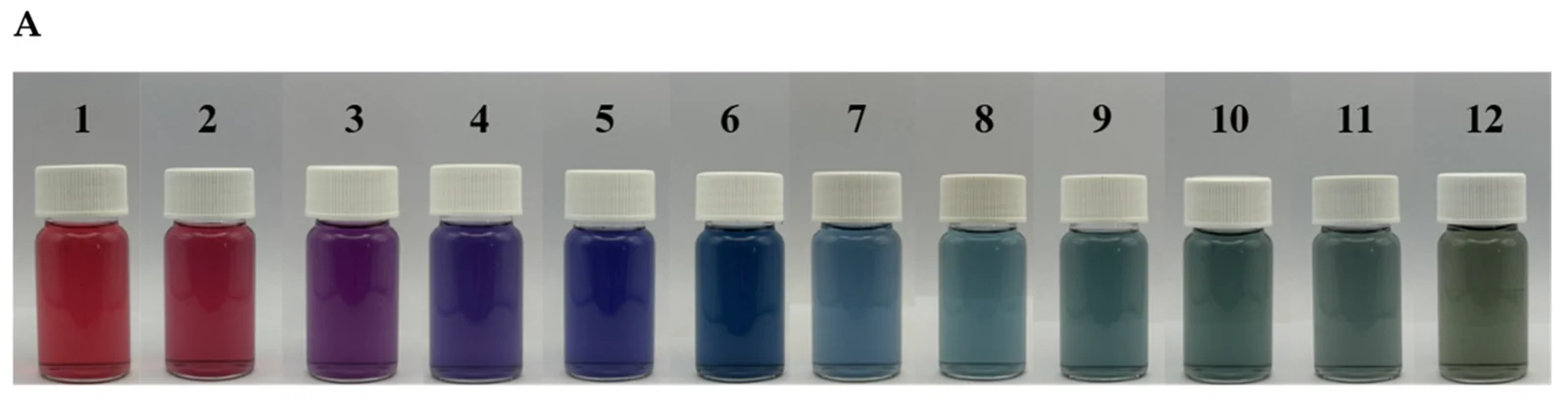

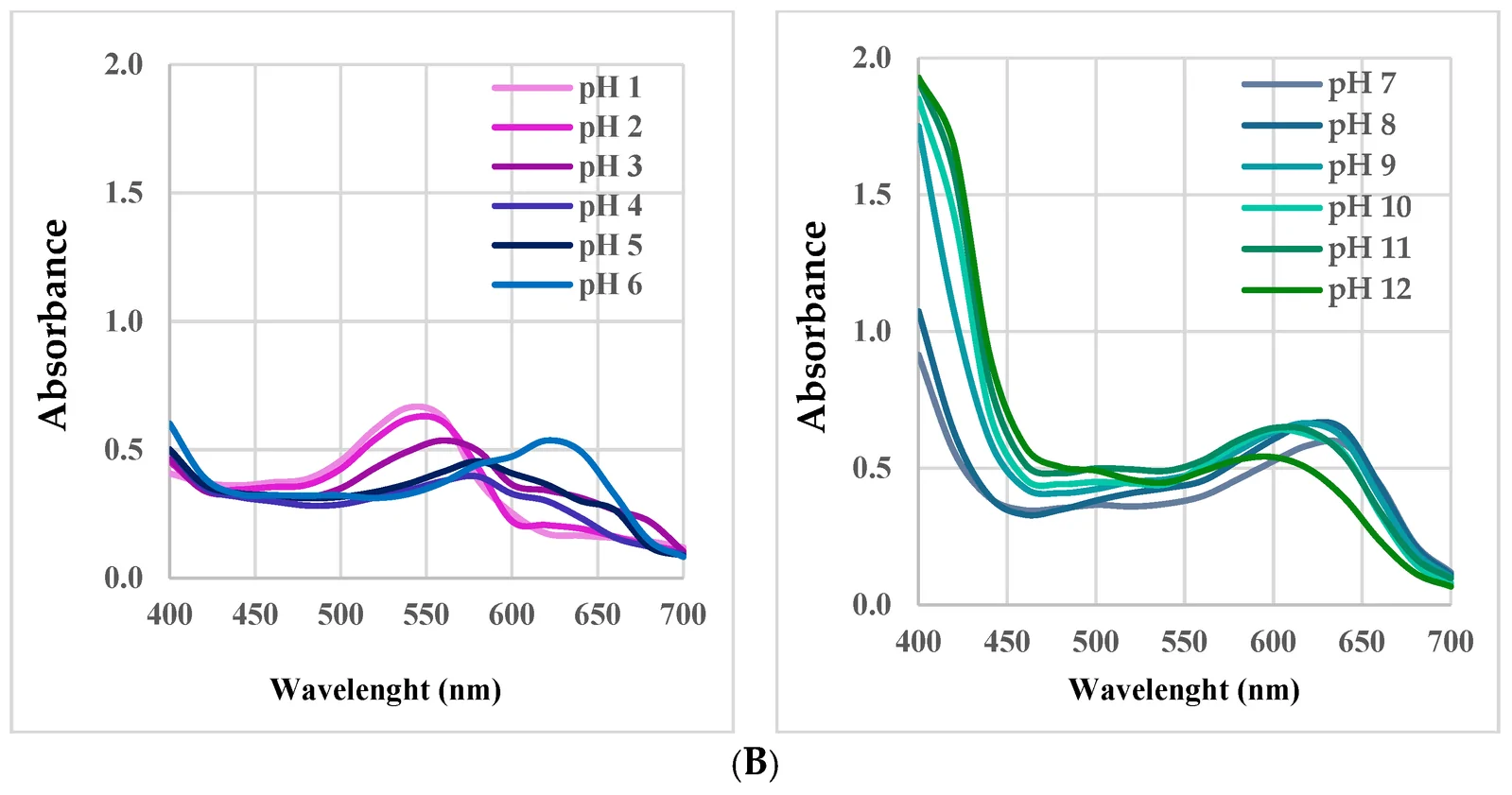
Visual color change in BPF powder in buffer solutions ranging from pH 1 to pH 12 (**A**) composed with UV–Vis absorption spectra (400–700 nm) for each pH (**B**).

Figure 1 shows the color changes and absorption spectra of butterfly pea flower (BPF) solution over a pH range of 1–12. As demonstrated (Figure 1A), under strongly acidic conditions (pH 1–2), the BPF solutions exhibited red to reddish-pink colors. At pH 3–5, the BPF solutions appeared purple to violet in color, while increasing the pH from 6 to 7 resulted in a gradual transition from dark blue to light blue. Under alkaline conditions (pH > 8), the BPF solution exhibited a shift from blue–green through turquoise to olive green. As observed, the BPF solution exhibited distinct color changes visible to the naked eye. Previous study also showed that changes in the color of the BPF extracts ranged from pink under acid conditions through purple, blue, green, and yellow under highly alkaline conditions [34,39]. According to Narayanan et al. [34] and Meshram et al. [39], these changes are attributed to pH-dependent structural transformations of anthocyanins. Under strongly acidic conditions, anthocyanins occur mainly as red flavylium cations. An increase in pH causes deprotonation and the formation of the purple quinoidal base, followed by the blue anionic quinoidal base. Under alkaline conditions, further structural transformations lead to the formation of yellow chalcone.

The color changes were also reflected in the UV-Vis spectra (Figure 1B). At pH 1–2, the main absorption peak occurred at approximately 542–546 nm. A further increase in pH resulted in a bathochromic shift, with λmax reaching 569–575 nm at pH 3–5, while at pH 6–8 the absorption maximum shifted to 625–629 nm. This bathochromic shift can be associated with anthocyanin deprotonation and the formation of quinoidal forms [40,41]. At pH values above 8, a gradual hypsochromic shift was observed, with λmax decreasing from 618 nm at pH 9 to 591 nm at pH 12. Moreover, at pH 9–12, higher absorbance was observed at 400 nm. This may result from the formation of yellow chalcone forms. Similar changes in the color and absorption spectra for butterfly pea anthocyanins have been reported by Ahmad et al. [40] and Fu et al. [41].

The anthocyanin present in butterfly pea flowers is predominantly characterized by polyacylated delphinidin derivatives, known as ternatins, which are responsible for the characteristic blue coloration of the flowers [28]. For example, Escher et al. [42] tentatively identified ternatin B4, ternatin B2/B3, ternatin D2, and other delphinidin derivatives in BPF extracts, suggesting that anthocyanins of this type may contribute to the pH-dependent color response observed in the present study.

### 3.2. Characteristics of Films

The physical and optical characteristics of the films are shown in Table 1. It was observed that the thickness of the pullulan films depended on the addition of BPF to the pullulan matrix. Finally, the thickness of the P_C film was 187.73 µm, and upon incorporation of BPF at 2% and 4%, the thickness gradually but not significantly increased to 190.63 and 200.56 µm, respectively. Similar observations were made by Santos et al. [35], who demonstrated that adding *C. ternatea* extract to sodium alginate films did not significantly affect film thickness. In turn, Qui et al. [43] indicated that adding the extract to the film significantly increased its thickness, but only at a concentration of 20%; with lower extract concentrations, this trend was not observed. The authors of the work point out that a lower extract concentration in films promotes better dispersion of the extract within the polymer matrix and the formation of a more compact film structure.

It was also observed that incorporating BPF imparted color to the pullulan films, and with increasing BPF content, the color of the films became more intense. The control pullulan films were characterized by high lightness (L* value) and the absence of coloration (low a* and b* values). The addition of BPF significantly reduced the L* value, thus that films become darker. As the BPF content in the pullulan films increased, the a* and b* values decreased significantly, which indicates a color shift toward green and blue, respectively. However, the decrease in the b* value was considerably greater than that in the a* value, indicating that the blue component had a greater influence on the final film color and corresponding to their blue appearance. This observation was consistent with previous studies showing that incorporating natural colorants into polymer matrices affects film color according to the characteristic colors of the incorporated pigments [35,44,45].

Additionally, the incorporation of BPF into the pullulan film matrix significantly influenced optical properties, including opacity and light barrier. The results show that as the amount of BPF increased, the film opacity increased from 0.271 for the control pullulan film to 2.06 and 3.29 for pullulan films containing 2% and 4% BPF, respectively. At the same time, a marked effect of BPF presence in the film was observed on the UV transmittance at 280 nm and the visible transmittance at 600 nm. The control pullulan film exhibited the highest UV and visible light transmittance, at 77.83% and 89.29%, respectively. The addition of BPF significantly reduced UV and visible light transmission through the pullulan films, and, moreover, a further decrease in transmittance was observed as the BPF content increased from 2% to 4%. Thus, the addition of BPF significantly increased the opacity and improved the light-barrier properties of pullulan films. This might be due to the presence of phenolic compounds, specifically anthocyanins, in the BPF. Wu et al. [46] reported that anthocyanins contain chromophoric structures with conjugated double bonds and aromatic rings that absorb radiation in the UV and visible regions. Thus, light absorption and scattering by anthocyanins may also affect film opacity [47]. Changes in the color and transparency of the tested films were also confirmed by their visual appearance, as shown in Table 1.

### 3.3. pH-Sensitivity and Ammonia-Sensitivity of Films

An important property of colorimetric films used for food freshness monitoring is their ability to respond to pH changes associated with food spoilage. Therefore, P + BPF_2 and P + BPF_4 films were evaluated for their sensitivity to different pH levels and ammonia conditions. The sensitivity of the films was evaluated based on visual observations and colorimetric measurements of color changes.

**Figure 2:**
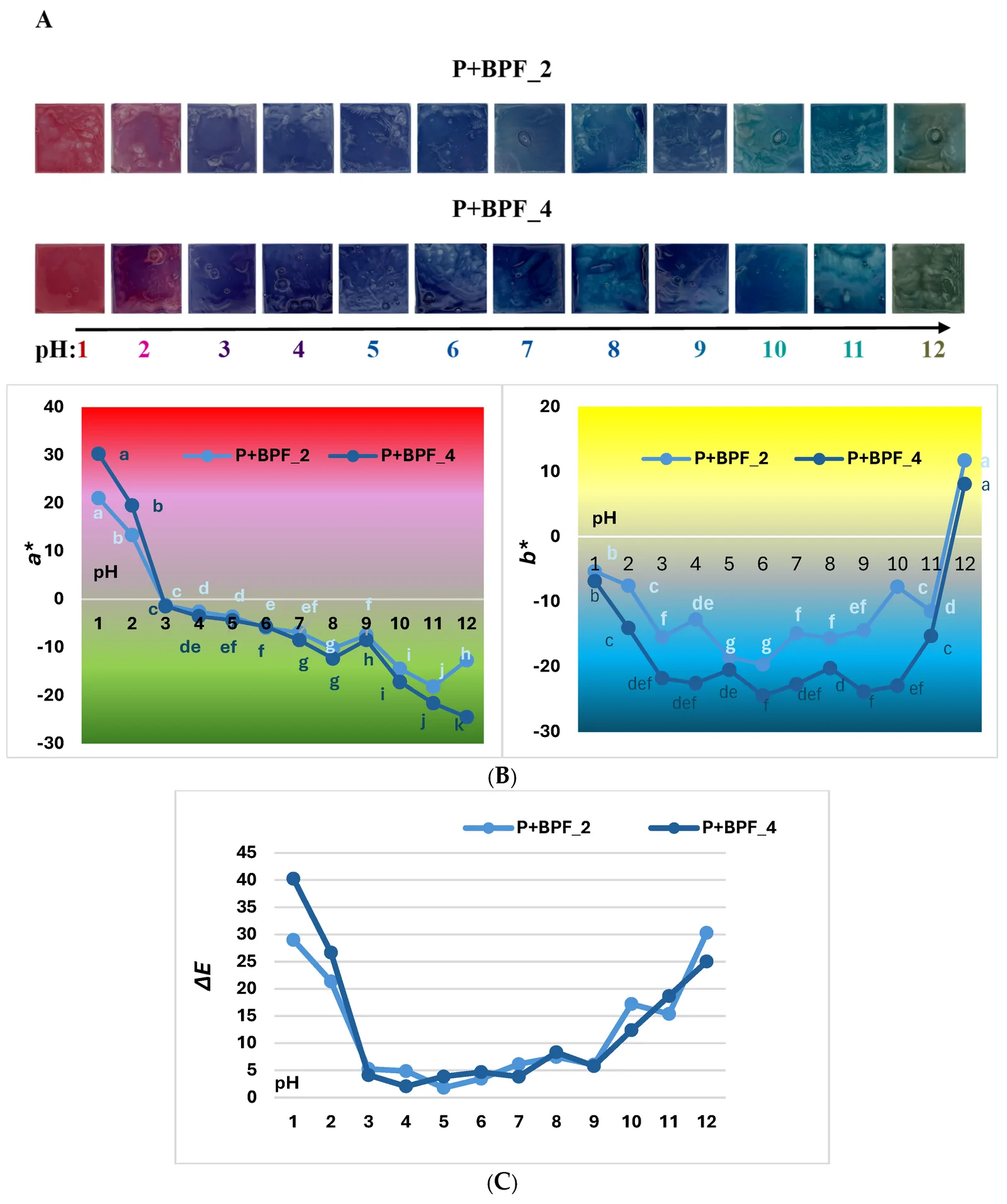
Color changes in films: P + BPF_2 and P + BPF_4 during exposure to different pH values; (**A**) visual changes in the film; (**B**) changes in color components a* and b*; (**C**) total color difference (Δ*E*). P + BPF_2—pullulan film with BPF at a concentration of 2%; P + BPF_4—pullulan film with BPF at a concentration of 4%. Different letters indicate statistically significant differences (*p* < 0.05) among pH values within each film.

Visual color responses of films to different pH show color variation as presents on Figure 2. Under strongly acidic conditions (pH 1.0–2.0), the films were reddish-pink, and their color changed from purple, violet to ocean blue with increasing pH (3.0–8.0). Under alkaline conditions (pH 9.0–12.0), the color gradually changed from turquoise to olive green (Figure 2A). These visual color changes in films were also reflected in the a* and b* values (Figure 2B). Under strongly acidic conditions, positive a* values (indicating red) were observed, which decreased with increasing pH and became negative (indicating green) under alkaline conditions. In contrast, negative b* values (indicating blue) decreased gradually as the pH increased, but under strongly alkaline conditions, they shifted to high positive values (indicating yellow).

Afterward, color changes between films exposed to various pH levels and the initial film were analyzed based on the Δ*E* parameter (Figure 2C). In general, Δ*E* values below 2 indicate no difference or only slight color difference that may be notice only by a trained observer; Δ*E* values ranging from 2 to 5 indicate color differences noticeable by an untrained observer, whereas Δ*E* values above 5 indicate a distinct difference between the compared colors, which can be recorded by naked eye. Therefore, for colorimetric indicators, Δ*E* values exceeding 5 are expected. This was also noted by Kaewprachu et al. [10] who found that a Δ*E* value above 5 observed for carboxymethyl cellulose films enriched with anthocyanins from Karanda and butterfly pea, indicated good visual color variability and allowed the films to be visually distinguished. The results of our study showed that Δ*E* values were above 5 for films exposed to strongly acidic conditions (pH 1–2) and alkaline conditions (pH 8 to 12). For films exposed to slightly acidic and neutral environments (pH 3–7), smaller color changes were observed, expressed by Δ*E* values of approximately 5. Previous research has shown results consistent with those obtained in the present study. For example, Asikkutlu and Yildirim-Yalcin et al. [48] observed visual color changes in avocado seed starch-based films containing butterfly pea flower extract at different pH values. The films changed color from pink under acidic conditions, through purple and blue, to green under alkaline conditions.

The sensitivity of P + BPF_2 and P + BPF_4 films to color changes under alkaline conditions indicates their potential use for monitoring the freshness of protein-rich food products, such as seafood and meat. During the spoilage of such products, microbial activity and protein degradation lead to the formation and accumulation of volatile nitrogen compounds, including ammonia and amines, which raise pH. Therefore, ammonia can be used as an indicator of changes associated with the spoilage of high-protein foods. To evaluate the color response of the films, they were exposed to ammonia at various concentrations and exposure times.

**Figure 3:**
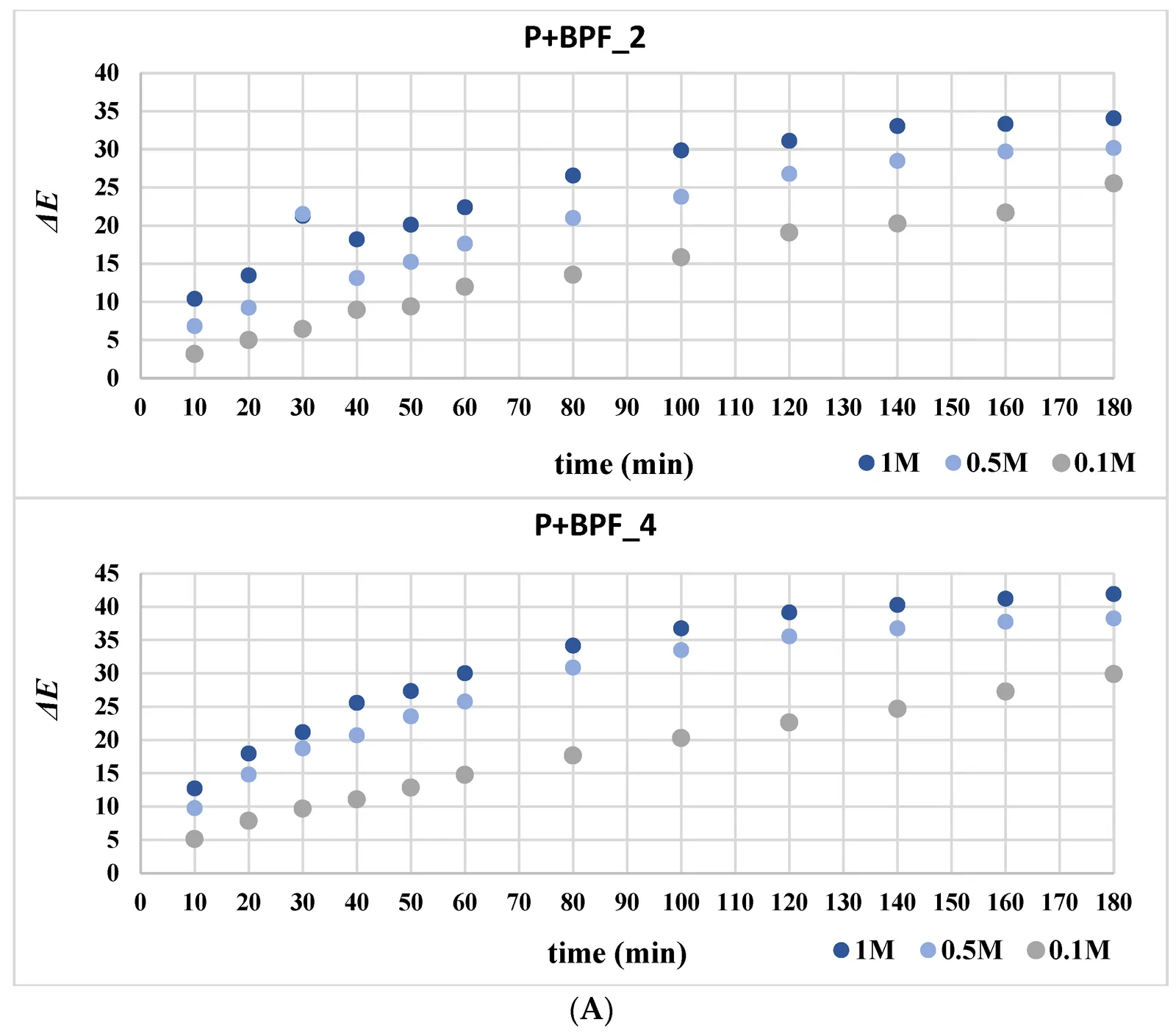

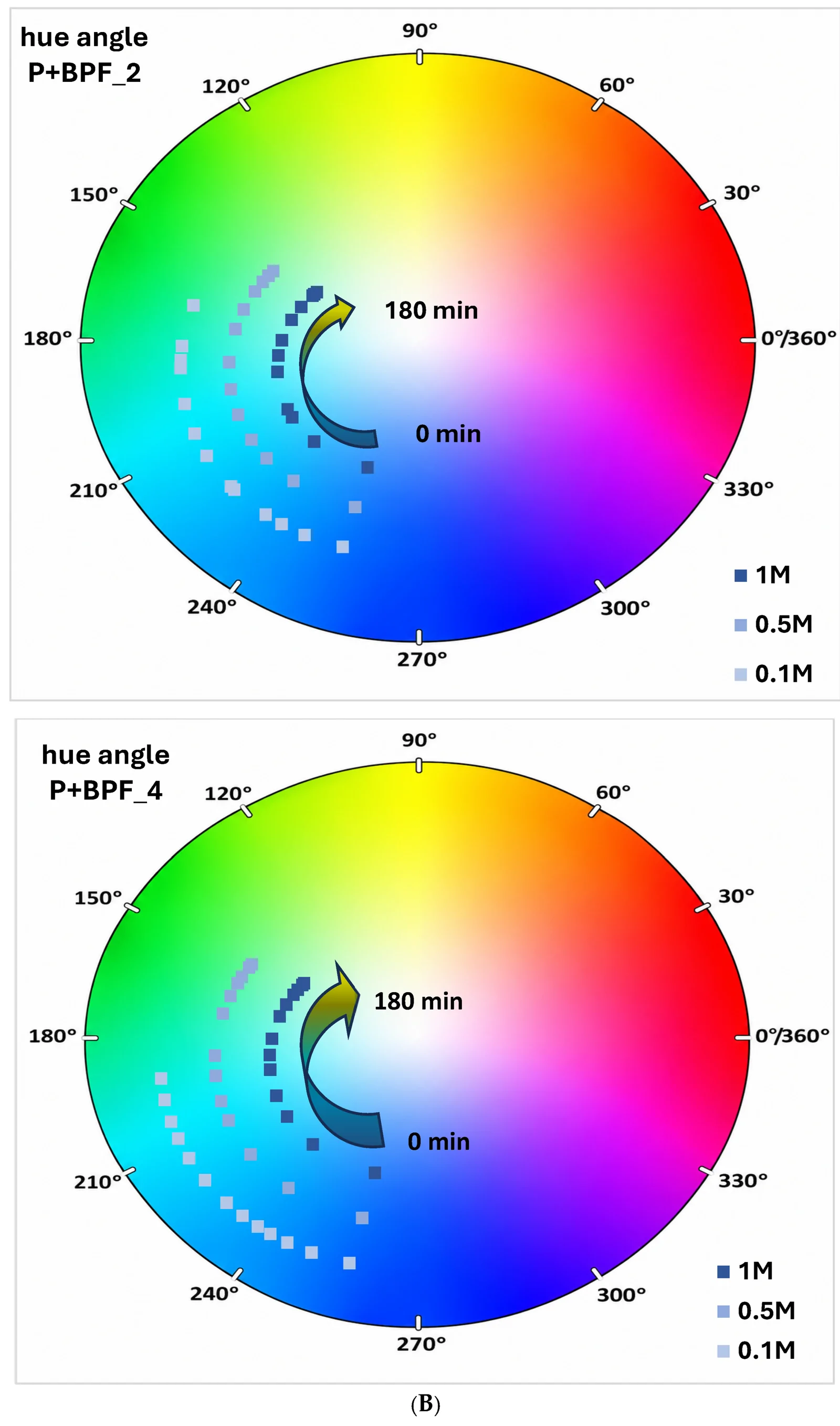

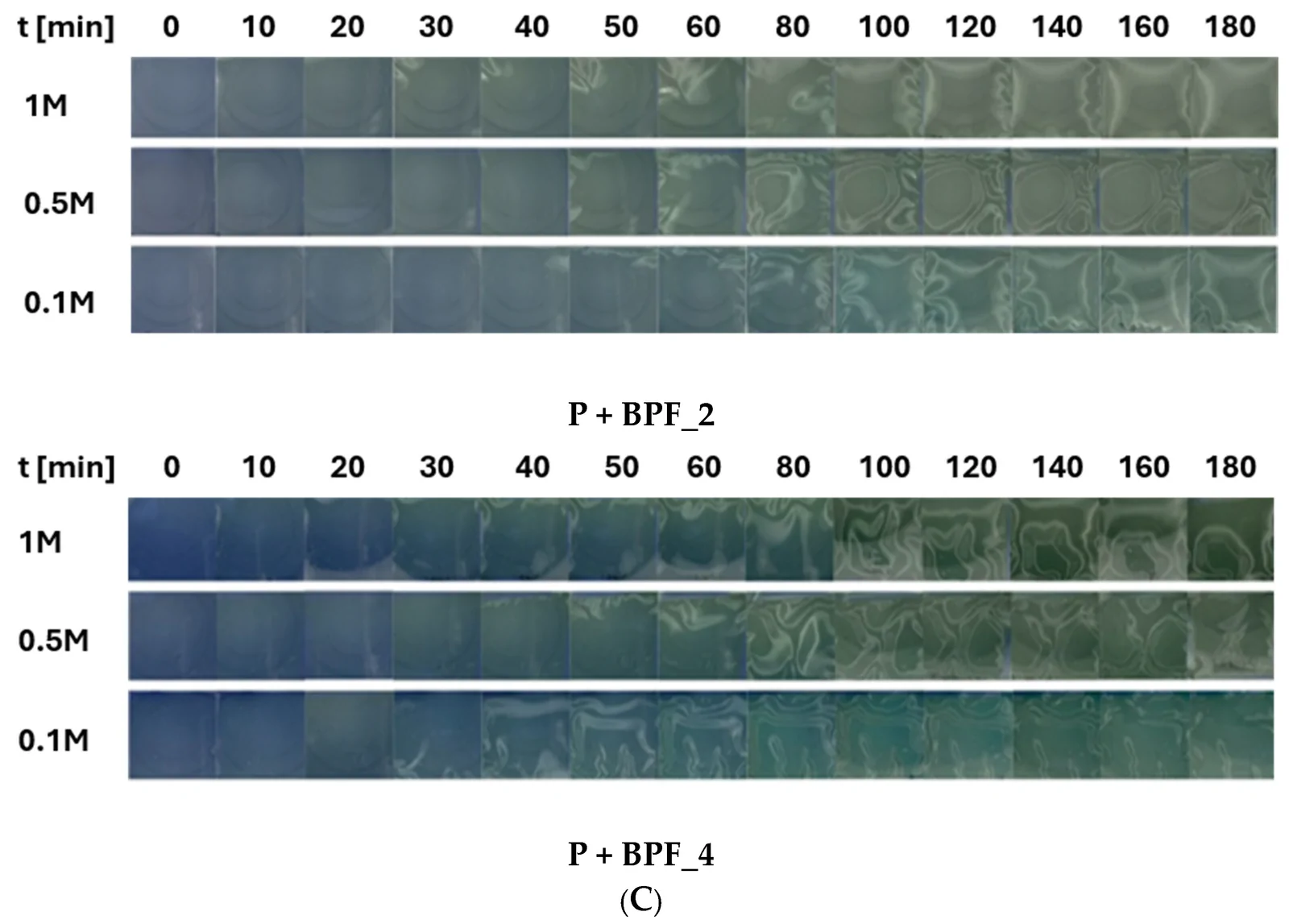
Color changes in films: P + BPF_2 and P + BPF_4 during exposure to different ammonia concentrations: (**A**) changes in total color difference (Δ*E*), (**B**) changes in hue angle (*h*°), and (**C**) visual changes in the film. P + BPF_2—pullulan film with BPF at a concentration of 2%, P + BPF_4—pullulan film with BPF at a concentration of 4%.

The color response of the P + BPF_2 and P + BPF_4 films was evaluated during 180 min of exposure to ammonia at concentrations of 0.1, 0.5, and 1 M (Figure 3A). As described above, a Δ*E* value above 5 was used as the criterion for determining the response time, indicating a clearly noticeable color change. It was shown that the films presented a visible response within 10 min of exposure at ammonia concentrations of 0.5 and 1 M, whereas a slower response was observed at 0.1 M. In the case of exposure to 0.1 M ammonia, the film with the addition of 2% BPF exhibited noticeable color changes after 20 min, whereas the film with 4% BPF showed such changes after 10 min. It was also observed that as ammonia concentration and exposure time increased, the Δ*E* values of the films gradually increased. However, higher Δ*E* values were recorded for the film containing 4% of BPF. Overall, the results indicate that the films exhibited noticeable color changes that could be visually distinguished from their initial colors.

To investigate the direction of color changes in the films upon exposure to ammonia, the hue angle (*h*°) was determined (Figure 3B). The *h*° value describes the relationship between the a* and b* components in the CIELab color space and allow the identification of the direction of color change. On *h*° scale, an angle of 0° and 360° represents a red hue, while angles of 90°, 180°, and 270° represent yellow, green, and blue hues, respectively [49]. The P + BPF_2 film showed a faster transition from the blue to the green hue region. This transition occurred after approximately 120 min at an ammonia concentration of 0.1 M and after approximately 40–50 min at ammonia concentrations of 0.5 M and 1 M, respectively. In contrast, the P + BPF_4 did not reach the green hue region within 180 min at an ammonia concentration of 0.1 M, whereas at 0.5 M and 1 M, the transition occurred after approximately 50–60 min. Overall, visually observed color responses of P + BPF_2 and P + BPF_4 films showed that the initial bluish color of the films gradually changed to olive green after exposure to ammonia (Figure 3C).

According to Ye et al. [50] the color changes in the film result from a reaction between volatile ammonia and water molecules within the film matrix. During this reaction, the formation of hydroxyl (OH^−^) and ammonium (NH_4_^+^) ions occurs. Eventually, the accumulation of hydroxyl ions leads to the alkalization of the film surface, promoting the deprotonation of the anthocyanins present in the film and the associated color change.

Based on visual observations (Figure 3C) and Δ*E* values (Figure 3A), P + BPF_4 appeared to exhibit more intense coloration and higher Δ*E* values than P + BPF_2 upon exposure to ammonia vapor, especially at higher ammonia concentrations. The importance of both visual color intensity and colorimetric response to ammonia was also highlighted by Khanjanzadeh et al. [51] who investigated PVA-based indicator films containing different amounts (0.1%, 0.3%, and 0.5%) of cellulose nanofibrils with immobilized neutral red. They observed that films with the lowest content (0.1%) showed faint colors despite rapid color changes upon exposure to ammonia vapor. In contrast, at the highest content (0.5%), the resulting color changes were barely noticeable to the naked eye. The authors suggested that this behavior could be related to the overlapping of dye pigments, leading to color darkening. Furthermore, films containing 0.1% and 0.5% of the cellulose nanofibrils with neutral red exhibited lower Δ*E* values than those containing 0.3%. These findings suggest that Δ*E* values may serve as one criterion for selecting an appropriate indicator film. Similarly, Miao et al. [52] selected an indicator film that exhibited the highest Δ*E* values in response to ammonia vapor for further shrimp freshness monitoring experiments, which supports the use of Δ*E* as one of the criteria for indicator film selection. In the present study, P + BPF_4 generally exhibited higher Δ*E* values than P + BPF_2, indicating a greater colorimetric response to ammonia vapor. Additionally, visual observations (Figure 3C) showed that P + BPF_4 exhibited more intense coloration upon exposure to ammonia. Therefore, given its higher Δ*E* values and more pronounced visual coloration, P + BPF_4 was selected for further monitoring of shrimp freshness.

### 3.4. Color Stability of Films

The color stability of P + BPF_2 and P + BPF_4 films was evaluated during 21 days of storage under different conditions: 4 °C in the dark (RH 70%), 23 °C under light exposure (RH 50%), and 23 °C in the dark (RH 50%) (Table 2). Generally, both films showed small changes in total color difference (Δ*E*). The lowest Δ*E* values were observed for films stored at 23 °C in the dark, where Δ*E* did not exceed 2. In contrast, films stored at 23 °C under light exposure and at 4 °C in the dark showed slightly higher Δ*E* values of 3.60 and 2.57, respectively, after 21 days of storage. However, the Δ*E* values did not exceed 5 for any of the tested films. Moreover, the hue angle (*h*°) values indicated that both films remained blue throughout storage. The effect of light on film color stability was also observed by Bao et al. [53], who reported lower Δ*E* values for films stored in the dark (below 3.16) than for those exposed to light (approximately 4.29) after 14 days of storage. However, in contrast to the present study, Hashim et al. [54] observed lower Δ*E* values for films containing anthocyanins from purple cabbage stored at 4 °C than at 25 °C. Similar observations were reported by Li et al. [55] for purple sweet potato-based films, with ΔE values below 2 and 3 at 4 and 25 °C, respectively, after 30 days of storage. Nevertheless, according to Wang et al. [56], films with Δ*E* values below 5 exhibit good color stability.

### 3.5. Application of Colorimetric Film in Monitoring the Freshness of Shrimp

**Figure 4:**
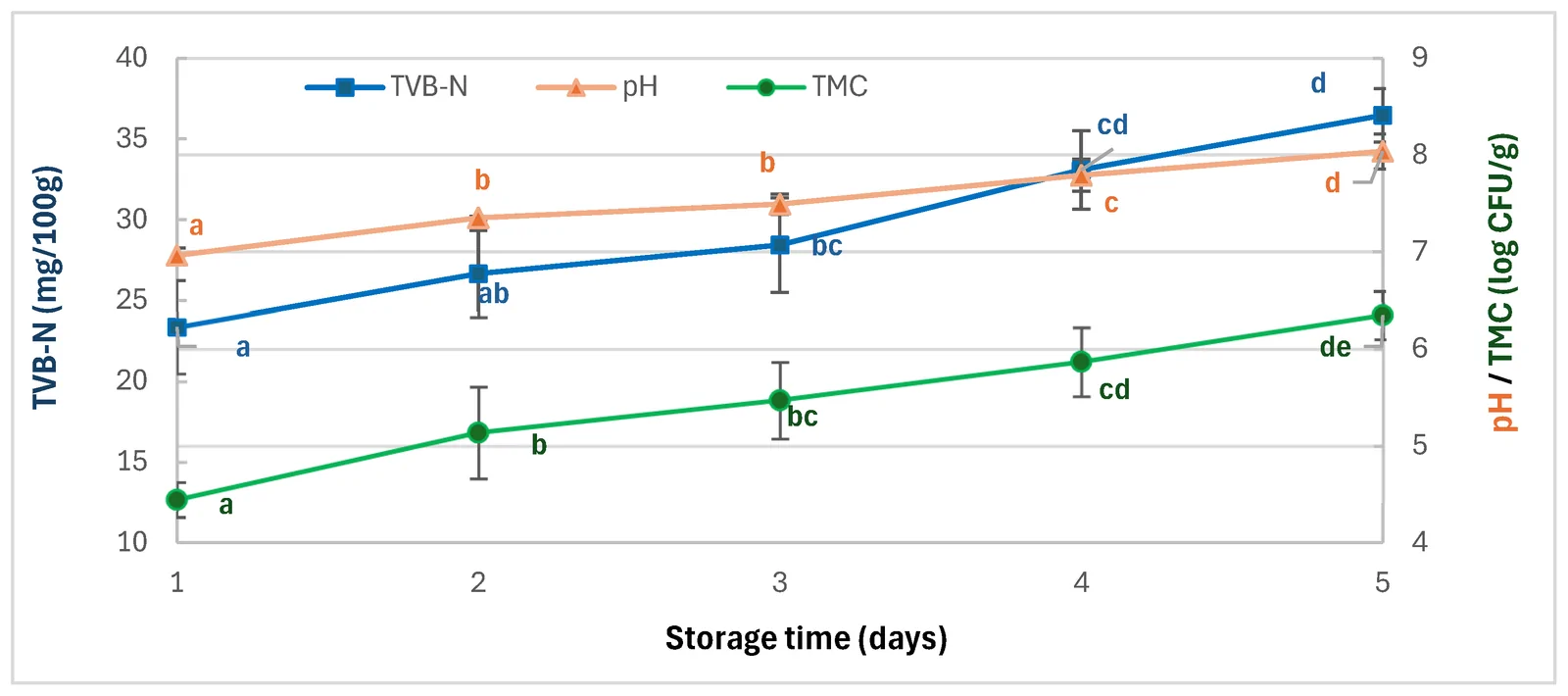
TVB–N, pH, and TMC of shrimp during storage at 4 °C for 4 days. Different letters indicate statistically significant differences between storage days for each parameter (*p* < 0.05).

**Figure 5:**
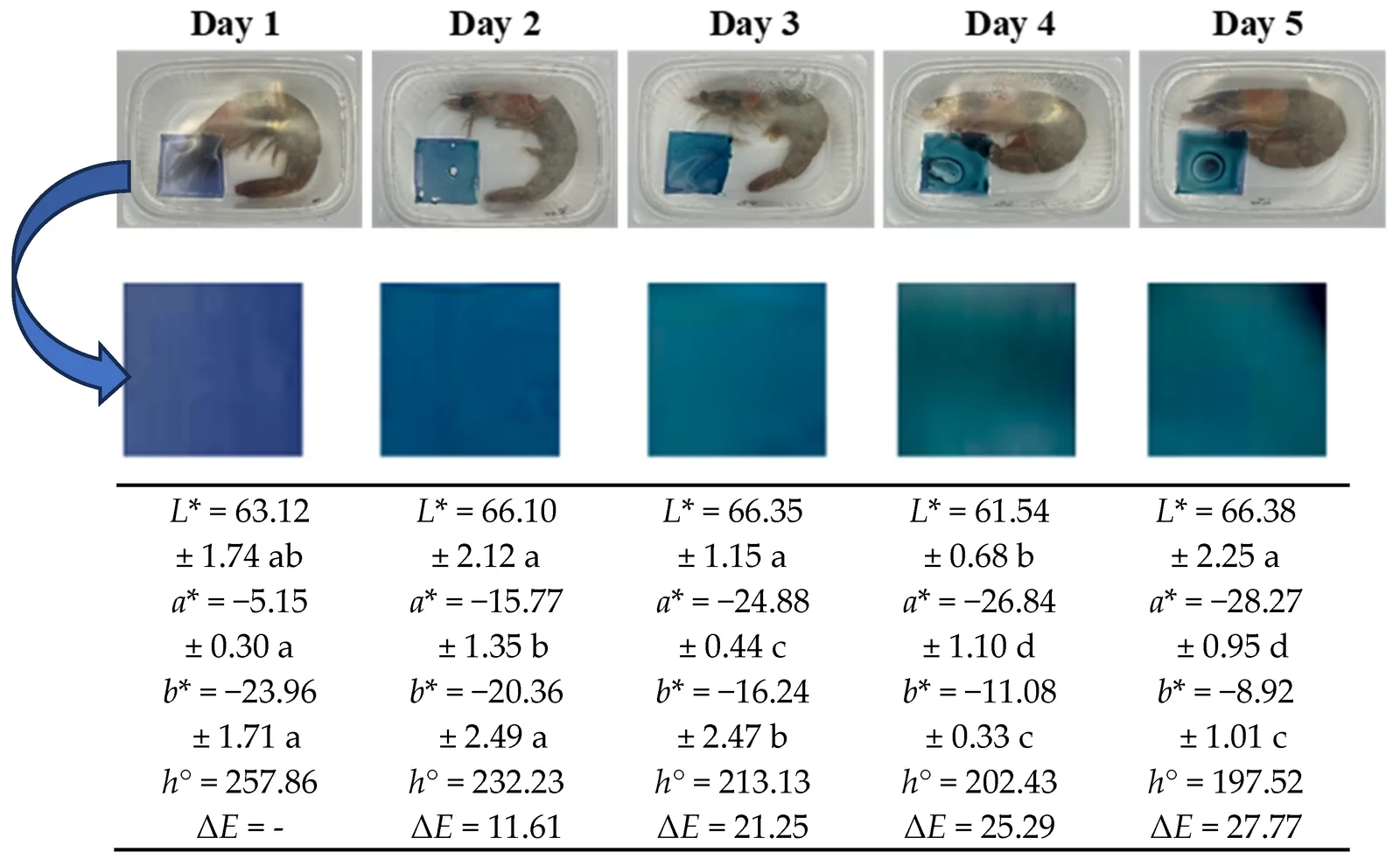
Visual and colorimetric color changes in the P + BPF_4 colorimetric film labeled on the package lid during the storage of shrimp at 4 °C for 5 days; a–d—different letters in the row indicate statistical differences (*n* = 5).

The final stage of this study was to evaluate the suitability of P + BPF_4 as a colorimetric film for monitoring shrimp freshness. During shrimp storage, the activity of microorganisms and proteolytic enzymes leads to the formation of volatile compounds such as ammonia and amines, included as components of total volatile basic nitrogen (TVB–N). The accumulation of these compounds raises the pH and produces a strong, unpleasant ammonia odor, indicating fish spoilage. Therefore, TVB–N is commonly used as a freshness index to assess shrimp quality [5]. In this context and based on results from films exposed to ammonia vapor, it was assumed that the accumulation of volatile basic nitrogen compounds during shrimp spoilage would induce a color change in the tested colorimetric film. Therefore, in this study, visual and colorimetric changes in the color of P + BPF_4 film were analyzed together with changes in TVB–N content, pH, and total microbial counts (TMC) in shrimp stored under refrigeration conditions (4 °C) for 5 days. The results regarding shrimp quality parameters are presented in Figure 4, while the color changes in the film used as a colorimetric label are shown in Figure 5.

The results of this study show that TVB–N content, pH, and TMC increase significantly during shrimp storage. At the beginning of shrimp storage, the initial values of TVB–N, pH, and TMC were 23.35 mg N/100 g, 6.97, and 4.44 log CFU/g, respectively. On day 5, at the end of storage, TVB-N, pH, and TMC reached 36.47 mg N/100 g, 8.04, and 6.35 log CFU/g, respectively. In general, shrimp are considered completely spoiled when TVB–N content exceeds 30 mg N/100 g, the pH value exceeds 7.7, and the microbial count is higher than 7 log CFU/g [57,58]. Based on these criteria, our study showed that the shrimp could be considered spoiled on day 4 of storage, when the TVB–N content reached 33.08 mg N/100 g, and the pH increased to 7.8, even though the bacterial count had not yet exceeded 7 log CFU/g. Moreover, results showed that on day 3, the TVB-N content had already approached the threshold value of 30 mg/100 g, indicating a high deterioration in shrimp quality and approaching the state of complete spoilage, while the pH and microbial count remained at considerably lower levels.

These changes in shrimp quality were associated with the film color response. As shown in Figure 4, the P + BPF_4 film visually changed color during shrimp storage, from blue recorded on days 1 and 2, through turquoise on day 3, to green recorded on days 4 and 5. These changes were also confirmed with colorimetric measurement. It was observed that a* values decreased, while b* values increased, and the hue angles (*h*°) of the film shifted from 257° to 197°, indicating that the initial bluish color of the film was changed to deep sea green with storage time. Furthermore, at each storage time, Δ*E* of the film was above 5, which indicates that the color change could be visually distinguished with the naked eye. In contrast, the films in the control packages without shrimp remained blue throughout the 5 days of storage. The Δ*E* value was 1.46, while the hue angle (*h*°) changed only slightly from 256.70° to 254.67°.

The potential of biopolymer-based films containing butterfly pea flower (BPF) anthocyanins for monitoring shrimp quality has been demonstrated in previous studies. For example, Kim et al. [59] developed gelatin/agar-based films containing BPF anthocyanins, which exhibited visible color changes from blue towards green during shrimp storage. Previous studies have also demonstrated that BPF-based indicators can respond to shrimp quality deterioration over a broad range of TVB–N values, from initially low levels to those associated with advanced spoilage. Kaewprachu et al. [10] investigated carboxymethyl cellulose-based films containing BPF anthocyanins during shrimp storage at 4 °C. The initial TVB–N content was 1.66 mg N/100 g and increased to 35.98 mg N/100 g after eight days at 4 °C. The film containing BPF anthocyanins changed from dark blue to dark teal during storage, demonstrating a color response to shrimp deterioration associated with spoilage. In contrast, Hashim et al. [60] demonstrated the applicability of agar-based films containing BPF extract for monitoring shrimp quality at 25 °C. In their study, TVB–N increased from 5.02 to 24.27 mg N/100 g over 24 h, while the film gradually changed from deep purple to blue at 3 h, bluish-green at 9 h, and greenish at 12 h, with the greenish coloration corresponding to shrimp spoilage according to the TVB–N criterion adopted by the authors.

In the present study, the potential of the P + BPF_4 film was evaluated using shrimp purchased from a local retail market. The initial TVB–N content was 23.35 mg N/100 g, indicating that monitoring began at a higher TVB–N level than in the studies discussed above. In contrast to previous studies conducted on shrimp with low initial TVB–N levels, this study demonstrates the potential of using the P + BPF_4 film to monitor further quality deterioration in commercially available shrimp with an elevated initial TVB–N content. However, due to the relatively high initial TVB–N content, the results of the present study cannot be used to assess the ability of the film to distinguish between different stages of shrimp quality deterioration, from very fresh to spoiled.

The Spearman correlation coefficients showed that Δ*E* was positively correlated with TVB-N, pH, and TMC, while *h*° was negatively correlated with these parameters (Table 3). These results indicate that the increase in shrimp spoilage parameters was accompanied by an increase in Δ*E* and a decrease in *h*° values of the P + BPF_4 film.

Although the results of this study indicate the potential of pullulan-based films with BPF powder to serve as colorimetric indicators for monitoring the freshness of shrimp, their practical application and commercialization require consideration of both technological and economic aspects. As highlighted by Asrafali et al. [8] the relatively high cost of biopolymers, including pullulan, represents an important economic challenge for their application in food packaging sector. The authors also highlight challenges associated with the uniform dispersion of functional additives and the reproducibility of film properties during large-scale production. Furthermore, they identify film drying as another technological challenge due to the risk of defects, such as cracking and non-uniform thickness, as well as the time and energy required for this process. This step can also be time-consuming and energy-intensive, increasing production costs [8]. These challenges highlight the need for further research to identify promising indicator materials that could be considered for future large-scale production.

## 4. Conclusions

The results of this study showed that BPF powder in concentrations of 2% and 4% can be effectively incorporated into a pullulan matrix to fabricate colorimetric films. The incorporation of BPF imparted a characteristic color to the films, increased film opacity, and improved their UV and visible-light barrier properties, but had no effect on film thickness. At the same time, a higher BPF content further increased opacity and enhanced blocking properties against UV and visible light but still did not significantly alter the film thickness. Both tested films demonstrated pH-dependent color changes and responded to ammonia vapor with a visually perceptible color change. Moreover, both films showed good color stability during 21 days of storage under the tested conditions. The final selected film, P + BPF_4, was successfully used in packaging as a colorimetric label for monitoring shrimp quality deterioration. It was demonstrated that the film changed color in association with shrimp spoilage. Although the color changes occurred gradually, the film showed potential for visually monitoring shrimp quality deterioration during refrigerated storage. However, further studies using shrimp with lower initial TVB–N values are needed to evaluate its use as an indicator of shrimp freshness, specifically to distinguish among stages such as fresh, moderately fresh, near-spoilage, and spoiled.

## Figures and Tables

**Table 1:** The physical and opacity characteristics of films.

	P_C	P + BPF_2	P + BPF_4
Thickness [µm]	187.73 ± 6.16 a	190.63 ± 11.18 a	200.56 ± 13.99 a
Color			
L*	94.93 ± 1.75 a	71.55 ± 1.31 b	64.89 ± 4.32 c
a*	0.05 ± 0.03 a	−4.24 ± 0.30 b	−5.42 ± 0.14 c
b*	0.41± 0.06 a	−16.88 ± 1.02 b	−22.68 ± 2.42 c
Opacity	0.271 ± 0.028 a	2.06 ± 0.18 b	3.29 ± 0.22 c
Transmission [%]			
280 nm	77.83 ± 0.96 a	0.43 ± 0.06 b	0.03 ± 0.008 c
600 nm	89.29 ± 2.13 a	42.95 ± 3.26 b	22.74 ± 0.64 c
Visual appearance of films	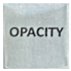	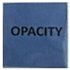	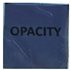

P_C—Control pullulan film without BPF, P + BPF_2—pullulan film with BPF at the concentration of 2%, P + BPF_4—pullulan film with BPF at the concentration of 4%, a–c—different letters in the row indicate statistical differences.

**Table 2:** Color stability of films under different storage conditions.

Temperature	Color Parameter	0 Day	7 Days	14 Days	21 Days
P + BPF_2					
4 °C dark (RH 70%)	Δ*E*	-	1.89	2.41	1.93
	*h*°	257.64	256.97	258.6	257.08
23 °C light (RH 50%)	Δ*E*	-	1.90	2.73	3.60
	*h*°	255.97	249.83	247.80	247.14
23 °C dark (RH 50%)	Δ*E*	-	1.04	1.61	1.70
	*h*°	255.66	255.36	255.24	253.70
P + BPF_4					
4 °C dark (RH 70%)	Δ*E*	-	1.24	2.91	2.57
	*h*°	256.18	256.96	254.95	255.55
23 °C light (RH 50%)	Δ*E*	-	2.20	3.19	2.89
	*h*°	259.47	259.49	260.42	259.93
23 °C dark (RH 50%)	Δ*E*	-	0.76	1.64	1.57
	*h*°	257.38	256.74	257.22	255.80

P + BPF_2—pullulan film with BPF at the concentration of 2% and P + BPF_4—pullulan film with BPF at the concentration of 4%.

**Table 3:** The Spearman correlation coefficient between the color parameters of the colorimetric film (calculated for the total color difference (Δ*E*) and hue angle (*h*°)) and shrimp spoilage indices TVB–N, pH, and TMC parameters.

Parameters	Spearman Correlation Coefficient	
	Δ*E*	*h*°
TVB-N	0.841	−0.885
pH	0.874	−0.934
TMC	0.767	−0.841

## Data Availability

The original contributions presented in this study are included in the article. Further inquiries can be directed to the corresponding authors.
